# Novel Nanocomposites of Carbon Nanomaterials and Poly(Neutral Red) Electropolymerized from Reline for DNA Damage Detection and Beverage Antioxidant Influence Assessment

**DOI:** 10.3390/bios15110735

**Published:** 2025-11-03

**Authors:** Anastasia Malanina, Rufiia Derbisheva, Tatiana Krasnova, Rezeda Shamagsumova, Vladimir Evtugyn, Alexey Ivanov, Anna Porfireva

**Affiliations:** 1 A.M. Butlerov’ Chemistry Institute, Kazan Federal University, 18 Kremlevskaya Street, Kazan 420008, Russia; 2 Interdisciplinary Center of Analytical Microscopy, Kazan Federal University, 18 Kremlevskaya Street, Kazan 420008, Russia

**Keywords:** neutral red, carbon nanomaterials, nanocomposite, deep eutectic solvent, reline, electropolymerization, DNA biosensor, voltammetry, DNA damage

## Abstract

Novel nanocomposites based on carbon black or multi-walled carbon nanotubes functionalized with carboxylic groups and Neutral red electropolymerized from reline were obtained in a one-step protocol and used for DNA biosensor development. The synthesis was carried out in potentiodynamic mode in a deep eutectic solvent reline consisting of a mixture of choline chloride and urea. The nanocomposite based on carbon black and poly(Neutral red) was applied for a voltammetric DNA biosensor developed to discriminate DNA damage. The sensor developed allowed the native, thermally denatured, and chemically oxidized DNA discrimination with either current changes or peak potential shifts. The nature of the DNA used had affected the sensor’s analytical response value. The DNA biosensor suggested was tested for the assessment of antioxidant capacity in such beverages as tea, coffee, white wine, and fruit-based drink purchased from local market. Simple, fast, and inexpensive approach of sensor modifying layer assembly would be demanded in control of food products and beverages quality, as well as for medical purposes.

## 1. Introduction

Recently, there have been a lot of scientific investigations devoted to DNA biosensors and their future development. These biosensors are widely used for genome decoding, kinship establishment, and genetic deceases, as well as for determination of pathogenic microorganisms and viruses [1,2,3,4]. Single-stranded DNA probes or aptamers are often used in such devices; herewith, the recognition is based on hybridization processes and complementarity principles. To provide the immobilization of ssDNA sequences, they can be either covalently linked or physically adsorbed at the transducer/modifier surface or entrapped into the polymeric matrix. For example, a Pt electrode modified with single-stranded DNA oligonucleotides entrapped within polypyrrole was used to detect bovine leukemia virus provirus DNA by electrochemical impedance spectroscopy and pulsed amperometric detection [5]. However, the investigation of DNA specific non-covalent interactions with low molecular analytes is not less important because such little molecules can be DNA damaging agents. Therefore, there is a necessity for DNA biosensors based on native double-stranded DNA (dsDNA).

Another trend of the investigations within novel DNA biosensors development is devoted to the novel approaches of the response recording, which do not suggest direct DNA electrochemistry. Polyelectrolyte complexes based on electropolymerized coatings playing the role of heterogeneous electron transfer mediators and dsDNA as the polyanion provide not only the biocomponent reliable immobilization but also DNA structure changes control due to the charge transfer redistribution, as well as the polymer coating properties [6].

**Figure 1:**
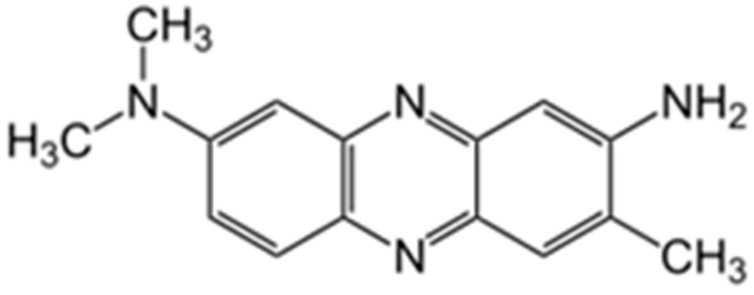
Chemical structure of NR dye.

Neutral Red (NR) is one of the most widely used electron transfer mediators. It is phenazine dye (Figure 1) capable of electrochemical polymerization, resulting in the electroactive coating formation.

The polymerization of NR as a representative of phenazine dyes is well-known and has been widely described in the literature [7]. The structure of the polymer formed strongly depends on the quantity of cation-radicals, which are generated when NR is oxidized, as these particles initiate the polymerization process and promote the branched chain obtaining [8]. The polymerization is going through amino groups participation and the following C-N binding was confirmed by infrared spectroscopy, Fourier transform Raman spectroscopy, and ^1^H nuclear magnetic resonance spectroscopy [9]. Polymeric films based on NR have been extensively using in the content of electrochemical sensors [10,11] and biosensors [12].

As the monomer’s limited water solubility resulted in low efficiency of the dye electropolymerization, the application of deep eutectic solvents (DESs) is an existing alternative to conventional solvents used for polymer synthesis [13]. The reason is its simplicity when using commercially available and natural components along with tunable physico-chemical properties. The perspectives of DESs application are quite different and provide their application in utterly different areas, such as organic synthesis and electrochemistry [14,15], for example, as extraction media [16], solvents for biological [17], and biomedical purposes [18]. As for the electrochemical sensors and biosensors development, using DESs within modifying coatings formation often leads to the improvement of both the coating characteristics and the required analytes determination parameters [19].

Presented in the literature examples of using NR polymeric films electrodeposited from DES media in the biosensors’ content confirm the prospects of such a class of solvents instead of aqueous media. DESs have effected not only on the monomers solubility degree but also on the morphology of the coating formed. As an example, the composite coatings based on Fe_2_O_3_ nanoparticles and poly(Neutral red) (PNR) electrodeposited from ethaline were used as a supporting layer for catalase immobilization and allowed to improve the limit of detection for hydrogen peroxide to 4.3 µM [20]. NR electropolymerization from ethaline on the surface of multi-walled carbon nanotubes (MWCNT) led to the coating peak currents increasing when compared to the polymeric film deposited directly onto the GCE surface. The simultaneous use of the carbon nanomaterials and DESs in the modifying layer content allowed for an increase in the sensitivity and has widened the concentration range for pyruvate and phosphate compared to the similar systems synthesized from aqueous media [21]. Layer-by-layer modification of GCE with porous graphite and PNR electrodeposited from DESs resulted in a five-times-higher voltammetric determination sensitivity towards hydroquinone [22].

**Figure 2:**
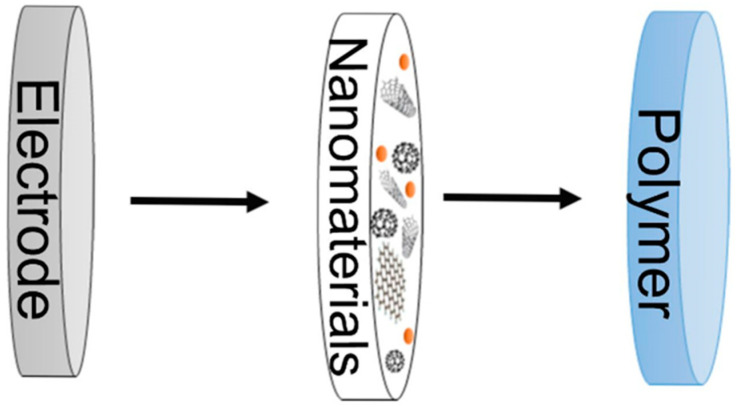
Scheme of modifying coating assembly based on nanomaterials and polymeric film.

The scheme of modifying layer assembly in all the systems considered above is shown in Figure 2. Nanomaterials in the coating content can increase the efficient electrode surface area, so that improving the modifying layer electrochemical parameters. However, as DESs have high dispersion ability, it seems more expedient to perform the electropolymerization of NR from DESs proximately in the presence of carbon nanomaterials.

In this pioneering work, DESs are suggested to be used as the medium for NR solubilization and carbon nanomaterials (CNM) dispersion simultaneously with the following electropolymerization, resulting in a nanocomposite of dye and carbon nanomaterial formation for the first time. Two types of carbon black (CB_250G_ and CB_350G_) were considered as such nanomaterials, as well as multi-walled carbon nanotubes functionalized with carboxyl groups (MWCNTf). Previously, the only work has demonstrated the application of DES for proflavine electropolymerization and electrochemically reduced graphene oxide nanocomposite formation during one-step synthesis [23]. The approach developed shows promising features of DES, which have never been investigated before. Reline is a binary mixture consisting of choline chloride and urea with a molar ratio of 1:2. Being the first representative of deep eutectic solvents studied [24], reline has been chosen as the electropolymerization medium. For the first time, the opportunity of resulting nanocomposites application in DNA biosensors assembly for voltammetric determination of DNA thermal or oxidative damage was established.

## 2. Materials and Methods

### 2.1. Reagents

NR (dye content 90%), urea, polystyrene sulfonate (PSS), and MWCNTf were purchased from Sigma-Aldrich (Saint Louis, MO, USA), choline chloride was from Acros Organics (Gell, Belgium), CB ENSACO 250 G (CB_250G_) and ENSACO 350 G (CB_350G_) were provided by Imerys (Willebroek, Belgium). Low-molecular DNA from salmon testes (DNAss) («Fluka», Steinheim, Germany) and high-molecular DNA from chicken erythrocytes (DNAch) (“Reanal”, Budapest, Hungary, average molecular mass 1.2 MDa) were used. Other reagents were of analytical grade and applied without any additional purification. Millipore Q deionized water (Simplicity^®^ water purification system, Merck-Millipore, Mosheim, France) was used to prepare working solutions. Voltammetric measurements were performed in 0.1 M phosphate buffer (PB) containing 0.1 M NaNO_3_, pH 7.0. The experiments with pH variation were carried out in 0.1 M PB or 0.04 M Britton-Robinson buffer (BRB).

### 2.2. Apparatus

Electrochemical measurements were performed with Metrohm DropSens µStat 400 Bipotentiostat/Galvanostat (Metrohm DropSens, Oviedo, Spain). Three-electrode system was presented as a screen-printed carbon electrode (SPCE). For the electrodes, printing DEC 248 printer (DEK, London, UK) and Lomond PE DS Laser Film (thickness 125 µm, Lomond Trading Ltd., Douglas, UK) were used. PSP-2 silver paste (Delta-Paste, Moscow, Russia) and carbon/graphite paste C2030519P4 (Gwent group, Pontypool, UK) were utilized as a base for the conductive tracks creation. The finishing insulating layer consisted of dielectric paste D2140114D5 (Gwent group, Pontypool, UK). For the electrodes printed hardening, they were treated at 80 °C. As a result, the three-electrode system obtained had the dimensions of 11 mm × 27 mm with a working electrode efficient surface area equal to 3.8 mm^2^ and a Ag pseudo-reference electrode. The electric contact between the electrode and the potentiostat was provided by the boxed connector (DropSens, S.L., Asturias, Llanera, Spain).

DropView 8400 Software (Metrohm DropSens) was applied to process the experimental data obtained.

Scanning electron microscopy (SEM) images of Metrohm DropSens DRP-110 SPCEs on the supports (DropSens, S.L., Asturias Llanera, Spain) modified with different coatings were obtained by a field emission scanning electron microscope of high resolution Merlin™ (Carl Zeiss AG, Oberkochen, Germany) in combination with AZtec X-Max.3 spectrometer (version 3.1). The software ZeissSmartSEM (version 6.06) was used for the image processing.

Statistical treatment of the results was performed using Microcal Origin 8.1 software pack.

### 2.3. Neutral Red Electropolymerization and DNA Biosensor Assembling

Reline was synthesized using ultrasonication according to the procedure described in our scientific group work recently [25]. Both the NR dye and CNM dispersion were performed simultaneously with the solvent synthesis. For this purpose, the exact amounts of the dye and/or CNM were added to the choline chloride and urea mixture before its homogenization with vortex and ultrasonication for 30 min. NR concentration in reline was equal to 0.1 M when CNM concentration was at the level of 1 mg/mL.

In case of NR electropolymerization only, after the synthesis, 100 µL of 0.1 M NR solution in reline was drop-cast onto the SPCE surface. The electropolymerization was performed in the potential range −1.2… +1.2 V at a scan rate of 0.15 V/s with 15 potential cycles. After the polymeric film (PNR_REL_) formation, the electrode was rinsed with distilled water, and the electrochemical stabilization of the surface was performed by ten-fold scanning of potential in a drop of 0.1 M PB (pH 7.0) within the potential range −1.2… +0.4 V at a scan rate of 0.15 V/s.

For the nanocomposite synthesis, 100 µL of 0.1 M NR and 1 mg/mL CNM mixture in reline were drop casted onto the SPCE surface. The electropolymerization and stabilization steps were performed in the same conditions as those in the absence of CNM.

For PNR_REL_ polymerization onto the CNM support, there was CB or MWCNTf dispersion in dimethylformamide (DMFA) prepared within 60 min of ultrasonication. After that, 2 µL of the dispersion prepared have been dried on the SPCE surface for 10 min at 60 °C. The surface was then rinsed with distilled water to wash unbound components and 100 µL of 0.1 M NR solution in reline was drop-cast onto the electrode surface for the following electropolymerization in the conditions mentioned above.

As NR solubility in PB is lower than that in reline, the dye concentration was 0.4 mM when it was electropolymerized from an aqueous medium. 100 µL of 0.4 mM NR solution in 0.1 PB, pH 7.0, was drop-cast onto the SPCE surface. Then the electropolymerization was carried out, followed by the stabilization of the coating formed in conditions similar to those in which PNR_REL_ had been obtained.

DNA immobilization was carried out with drop casting its followed by physical adsorption. For this purpose, 2 µL of 1 mg/mL DNA was drop-cast onto the electrode surface, covered with an Eppendorf tube for 10 min, then rinsed with distilled water and dried in the air. Thermally denatured or chemically oxidized DNA was included into the sensor content similarly. Thermal denaturation of DNA (DNA_den_) was performed as follows: the pristine solution was heated at 90 °C for 30 min and then rapidly cooled in ice for 5 min.

To obtain the solutions of chemically oxidized DNA with reactive oxygen species (ROS), there were various oxidizing agents used. Firstly, 1 mg of DNA was added to 1.2 mM H_2_O_2_ for 1 h (DNA_ox(H2O2)_). Also, ROS have participated in oxidation with the mixture of 0.4 mM Cu^2+^ and 15.2 mM H_2_O_2_ or with Fenton reagent, resulting in the formation of DNA_ox(Cu)_ or DNA_ox(F)_, respectively. For the last one, DNA was further treated with the mixture containing 0.4 mM disodium ethylenediaminetetraacetate dihydrate (EDTA), 2 M NaOH, 0.1 mM FeSO_4_, 0.4 mM ascorbic acid, and 0.9 mM H_2_O_2_ for half an hour.

For antioxidant protective effect assessment, the commercially available tea sachets, coffee, and cocoa samples were prepared according to their manufacturer’s recommendations. The sample of effervescent Vitamin C was prepared with the dilution of half of a tablet (450 mg of vitamin C) in 100 mL of distilled water. White vine and fruit-based drinks were used without any additional pretreatment. The samples were cooled to room temperature if necessary, and a 100 µL aliquot was collected into Eppendorf tube and diluted to 1 mL volume with distilled water.

The oxidation of DNA in presence of antioxidants was carried out in two methods. In the first one, the aliquot of the sample (25–150 µL) was previously mixed with the DNA solution for 20 min, and then H_2_O_2_ was added to the mixture. The second case suggested that the aliquot be previously mixed with H_2_O_2_ solution and added to the DNA after 20 min. In both cases the components ratio was calculated to reach the resulting oxidized DNA solution volume equal to 1 mL. After all the components were mixed, the solution was kept for 60 min, and then it was drop-cast onto the nanocomposite coating formed.

## 3. Results and Discussion

### 3.1. Electropolymerization of Neutral Red on Screen-Printed Carbon Electrodes from Phosphate Buffer or Reline

**Figure 3:**
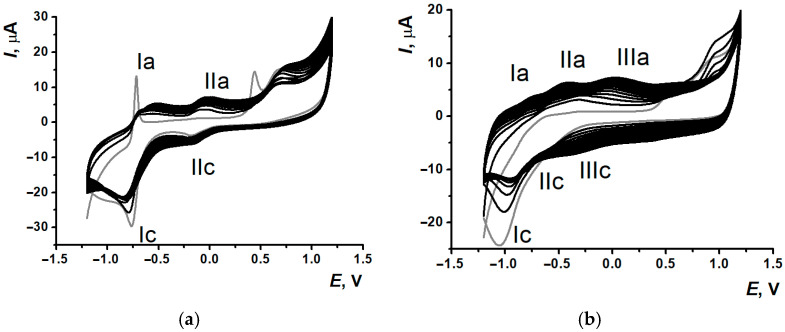
Multiple cyclic voltammograms of NR on SPCE (**a**) in 0.1 M PB, pH 7.0, and (**b**) in reline, 15 cycles, gray curve—the 1st cycle.

Within the potential multiple scanning for NR in PB and reline (see the conditions in Section 2.3), there were significant differences in the morphology of the voltammograms recorded (Figure 3).

Conveniently, there is a well-resolved pair of peaks related to both monomeric and polymeric forms of NR about −0.5 V and weakly evident peak pair at 0 V corresponded to doping-dedoping processes of the PNR on glassy carbon electrode [7]. When NR was electrodeposited on SPCEs from 0.1 M PB, pH 7.0 (further denoted as PNR_PB_), there were two pairs of well-pronounced redox peaks obtained on the voltammograms (Figure 3a). One of them (Ia/Ic) related to redox processes of phenazine core of monomeric and polymeric forms of the dye (~−0.6 V). As the second pair of peaks (IIa/IIc) occurred at the 2nd potential cycle, it was suggested to indicate the process of polymeric film formation, which could be referred either to oxidation and reduction of nitrogen in the bridges between polymer units [26] or to polymer doping-dedoping effects [7]. Also, splitting of the irreversible peak in the anodic potential area was observed at the 1st cycle (about 0.5 V and 0.7 V). The prior peak formation was provided by high degree of the dye adsorption onto the SPCE surface. Contrary to the electropolymerization onto GCE, it was the reason for the initial irreversible oxidation of NR adsorbed and the following diffusionally controlled oxidation with formation of the radicals providing the monomer polymerization.

Reline as a medium for the electropolymerization promoted the resolution of peaks for monomeric (at −1.0 V) and polymeric (at −0.7 V) forms of the dye (Figure 3b), so there were three pairs of peaks in the voltammograms of NR electropolymerization.

**Figure 4:**
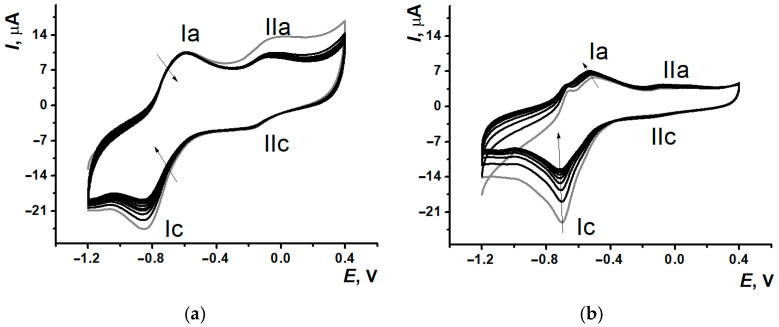
Signal stabilization (10 cycles) in 0.1 M PB, pH 7.0, for the coatings SPCE/PNR_PB_ (**a**) and SPCE/PNR_REL_ (**b**); gray curve—the 1st cycle, the arrows show redox peaks changing with the cycle number.

After PNR_PB_ and PNR_REL_ film formation on the SPCE surfaces, 100 µL of PB were drop casted onto the modified electrodes with the following electrochemical stabilization (See the protocol in Section 2.3). The voltammograms obtained (Figure 4) have demonstrated the significant decrease in (Ic) currents within the first 5–6 cycles of scanning with the following gradual stabilization, whereas the second pair signal (IIa/IIc) stayed quite stable.

Such a change of signal is conventional for polymeric coatings based on the dyes of the phenazine or phenothiazine group and provided by the release processes of the dye monomer units adsorbed on the electrode surface or entrapped into the polymeric film formed. The following assessment of the electrochemical characteristics of the studied coating has included both pairs of redox peaks.

### 3.2. Comparison of Electrochemical Properties of Poly(Neutral Red) from Reline and Phosphate Buffer

The dependencies of PNR_PB_ and PNR_REL_ redox peak potentials and currents on pH and scan rate were studied, as well as the according electrochemical characteristics have been established and compared. The coating stabilization by ten-fold potential scanning was utilized before the redox peaks recording.

Bilogarithmic dependencies log*I*_p_–log*v* of peaks pair (Ia/Ic) for both the coatings had slope values close to 0.5 (Table 1), indicating quasi-diffusional control of the redox reaction.

The second pair of peaks was more sensitive to the changes in the electropolymerization medium. PNR_PB_ electron transfer processes at 0 V have demonstrated the mixed diffusion-adsorption character, whereas PNR_REL_ had the slope of (IIa/IIc) peaks pair bilogarithmic dependencies being close to 1, which indicates the adsorption control. The appropriate linear regression equations are given in Table 2.

**Figure 5:**
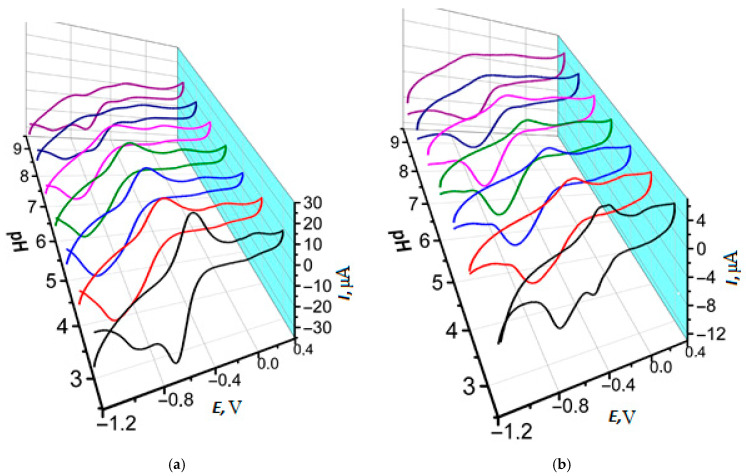
Cyclic voltamograms of SPCE/PNR_PB_ (**a**) and SPCE/PNR_REL_ (**b**) depended on pH of 0.1 M PB. (black) pH 3.0, (red) pH 4.0, (blue) pH 5.0, (green) pH 6.0, (magenta) pH 7.0, (dark blue) pH 8.0, (purple) pH 9.0.

The experiments with pH varying were carried out in PB and BRB to assess the influence of the ions’ nature. pH changing had a similar effect on the voltammograms morphology of polymeric coatings PNR_PB_ and PNR_REL_, though PNR_REL_ peaks in BRB were less defined (Figure S1). Cyclic voltammograms (Figure 5) showed the peak potential shift towards the cathodic area when pH increased. It was suggested that within the reduction process, the electron transfer must be preceded by protonation, and within the oxidation process, the polymeric film deprotonation took place. In addition, pH changes also had an effect on polymeric film peak currents. In a strongly acidic medium, the maximal peak currents were observed for the (Ia/Ic) pair of peaks, which were decreasing with the following pH increase. All the coatings have demonstrated the splitting of (Ic) peak and its current decreased in strongly acidic and strongly alkali media that could be provided by the simultaneous presence of mono- and diprotonated dye forms, reducing at different potentials.

Half-sum of the potentials of the PNR peaks pair was considered as the equilibrium redox potential and noted as *E*_m_. For the PNR_PB_ (Ia/Ic) peaks, there was a dependency of *E*_m_—pH with two inflections at pH 5.0 and 7.0 (Figure S2). These inflections were provided by the various forms of neutral and protonated dye involvement in the processes that could affect the resulting pH-dependencies slope [27]. Similar changes could be seen at the pH-dependencies obtained for PNR_REL_, although they were less obvious. At the same time, both the coatings in BRB demonstrated only one inflection at pH 5.0 for PNR_PB_ and at pH 7.0 for PNR_REL_ (Figure S3). As it is shown for the characteristics of *E*_m_—pH dependencies (Table 3), the slope values for (Ia/Ic) PNR_PB_ and PNR_REL_ in acidic medium differed almost twice, but the slope values became much closer to each other in alkali medium. At the same time, the slope for PNR_REL_ had almost four times decreased compared to PNR_PB_, when pH was changing from 2.0 to 5.0 (Table S1). The slope of PNR_REL_ pH dependency in weakly acidic media overrated that of PNR_PB_. As for alkali media, only PNR_PB_ had pronounced pH-dependency in that pH range.

### 3.3. Electrodeposition of Poly(Neutral Red) from Reline with Carbon Nanomaterials

In recent decades, CNMs have been widely used in the content of different modifying composites for biosensor development. Using such materials provides more rougher surface and improves electron transfer conditions. Mainly, CNMs are added by the drop-casting method using the dispersions after previous ultrasonication. In this work, CB and MWCNTf played the role of CNM.

Layer-by-layer PNR_REL_ deposition onto the CNM support led to a decrease in redox peak currents of polymeric dye phenazine fragment (Figure 3b and Figure S4). A significant background current increase was observed in the case of using CB_350G_ and MWCNTf supports (Figures S4 and S5). Anodic peak currents increased only for the peak pair (IIa/IIc) (Figure S6B). Using CB_250G_ as a supporting layer led to a significant increase in peak currents for both pairs (Figure S6). Therefore, using CNM as a supporting layer for NR electropolymerization had low efficiency. In addition, when the polymer was electrodeposited onto the supporting layer of CB_350G_ and MWCNTf, the standard deviation of currents recorded was more than 30%.

As an alternative approach to developing the nanocomposite material based on PNR and CNM, simultaneous electrodeposition of CNM and PNR was suggested. Such method can allow to reduce the development steps number, the volume of solvents used and the electrode modification period. CNM dispergation in reline was performed within its ultrasonication for 30 min. Time-stable dispersions were obtained only when CB was used, whereas dispergation of MWCNTf in DESs led to heterogeneous spumy mixtures obtained with its following splitting.

**Figure 6:**
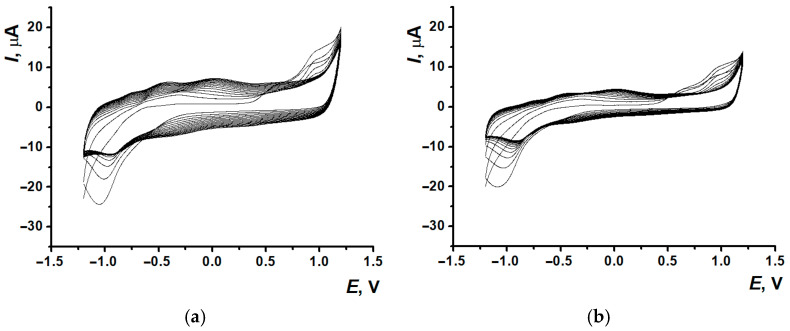

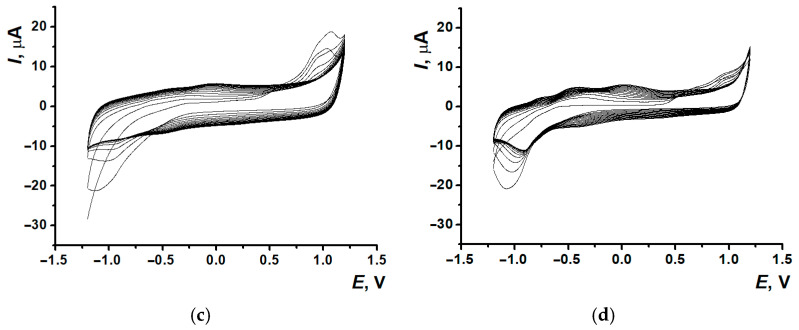
Voltammograms of NR electropolymerization in reline without CNM (**a**) and in the presence of CB_250G_ (**b**), CB_350G_ (**c**), MWCNTf (**d**).

Including CB_250G_ (Figure 6b) into the mixture for electropolymerization led to a rapid decrease in peak currents; however, the voltammogram’s morphology stayed the same. Using CB_350G_ (Figure 6c) in the composite promoted the growth of cation radical peak formation and its shift towards anodic area for 0.1 V. Nevertheless, the worst resolution of single polymeric forms was observed because of the polymeric coating broadened signal recorded after the 10th cycle. MWCNTf inclusion also resulted in a decrease in the currents recorded (Figure 6d).

**Figure 7:**
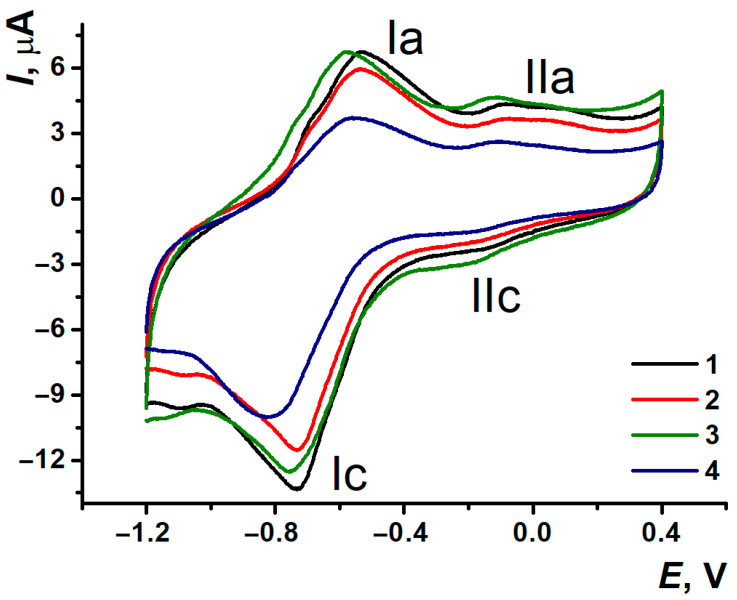
Cyclic voltammograms recorded on (1) SPCE/PNR_REL_, (2) SPCE/PNR_REL_(CB_250G_), (3) SPCE/PNR_REL_(CB_350G_), (4) SPCE/PNR_REL_(MWCNTf) after stabilization in 0.1 M PB, pH 7.0.

Two pairs of separated peaks were also observed when the electrodes modified with nanocomposites were transferred into a fresh portion of 0.1 M PB with no monomer (Figure 7).

**Figure 8:**
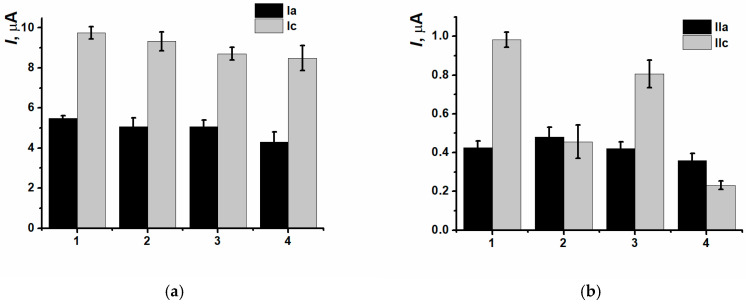
(Ia/Ic) (**a**) and (IIa/IIc) (**b**) peaks currents comparison for the (1) SPCE/PNR_REL_, (2) SPCE/PNR_REL_(CB_250G_), (3) SPCE/PNR_REL_(CB_350G_), (4) SPCE/PNR_REL_(MWCNTf).

CNM inclusion into the polymeric film led to insignificant decreasing of currents of (Ia/Ic) peaks pair, as well as for the nanocomposites based on CB_250G_ and MWCNTf (IIc) redox peaks currents decreasing was also observed (Figure 8).

Efficient surface area for the modified electrodes was assessed using the Randles-Sevčik Equation (1).

(1) In=(2.69×105)n32AcD12υ12

where *I*_p_ is oxidation peak current (A), *n*—electrons number, *A*—electrode efficient surface area (cm^2^), *c*—concentration (mol·cm^−3^), *D*—diffusion coefficient (cm^2^·s^−1^) and *υ*—potential scan rate (V·s^−1^). For K_4_[Fe(CN)]_6_ in 0.1 M KCl, *T* = 298 K, *n* = 1, *D* = 7.6 × 10^−6^ cm^2^·s^−1^ [28].

The formation of PNR_REL_ polymeric film on the SPCE led to an efficient surface area increase (2.46 ± 0.13 mm^2^) compared to the unmodified working electrode surface (1.64 ± 0.18 mm^2^). Efficient surface area was calculated in according to Equation (1) being equal to 2.34 ± 0.040 mm^2^ for SPCE/PNR_REL_(CB_250G_), 3.56 ± 0.078 mm^2^ for SPCE/PNR_REL_(CB_350G_) 3.56 ± 0.078 mm^2^, and 2.19 ± 0.159 mm^2^ for SPCE/PNR_REL_(MWCNTf), respectively.

**Figure 9:**
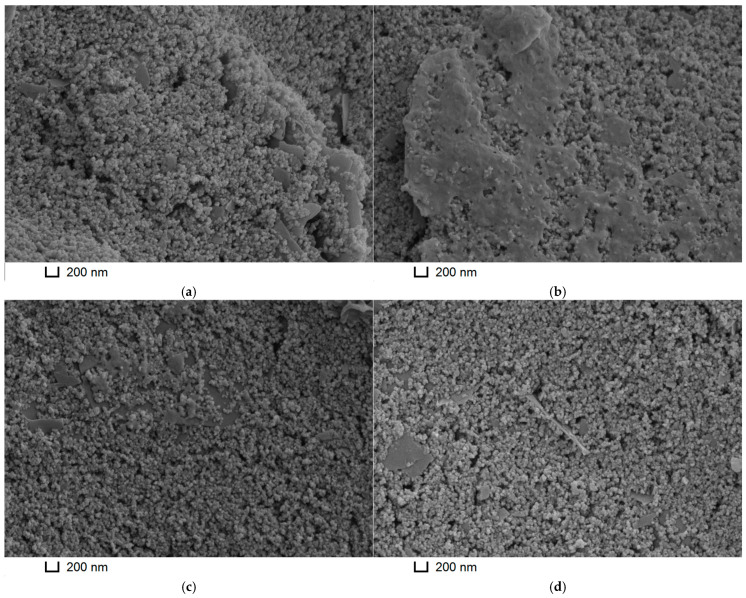
SEM images of the (**a**) PNR_REL_, (**b**) PNR_REL_(CB_250G_), (**c**) PNR_REL_(CB_350G_), and (**d**) PNR_REL_(MWCNTf) coatings.

The results obtained correlated with the morphology of the composites studied by the SEM method (Figure 9). PNR_REL_ and PNR_REL_(MWCNTf) looked like microgranulated coatings, and for PNR_REL_(MWCNTf), single nanotubes could hardly be differentiated from the polymeric grains. The inclusion of CB_250G_ into the composite content resulted in vast smoother bulk glaze-like sites. Agglomeration of single polymeric grains into dense glaze coatings explained electron transfer processes, as well as polymerization peak currents decreasing. When CB_350G_ was included in the composite, there was also partial agglomeration of the grains, which formed little smooth glaze-like sites, well noticeable in the left upper angle of the microimage. However, in this case, they were fairly evenly distributed on the surface and did not interfere with the electron transfer process. In latter case, such sites could act like microelectrodes array providing the biosensor analytical characteristics improved.

### 3.4. DNA Implementation Effect

Biocomponent immobilization is an important step in biosensor development. To immobilize DNAss onto the composite coatings based on PNR_REL_ and CNM, the drop casting method was chosen, in which the biopolymer was accumulated onto the polymer coating due to physical adsorption. For this purpose, 2 µL of 1 mg/mL DNA solution was drop-cast onto the modifying coating for 10 min.

**Figure 10:**
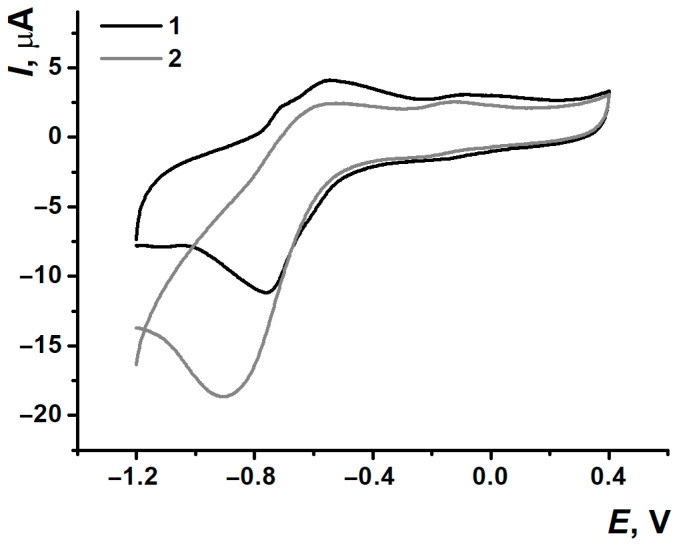
DNAss implementation influence on the cyclic voltammogram morphology, (1) SPCE/PNR_REL_ and (2) SPCE/PNR_REL_/DNAss.

Biopolymer drop casting has significantly changed the morphology of the voltammograms recorded (Figure 10). For the peaks pair (Ia/Ic), the resolution of the oxidation peak was worsening while the redox peaks were increasing. As for the second pair (IIa/IIc), the peak currents were decreasing after the exposition in DNAss solution, which could be conventionally related to the inclusion of non-conductive DNA molecules and electron transfer conditions.

Such a response change could be provided by the redistribution of polymeric grains at the electrode surface. The drop casting method for DNA immobilization did not allow for the creation of a uniform and homogeneous coating. This could lead to the partial “tightening” of positively charged PNR grains to the sites of negatively charged phosphate groups of DNAss accumulated.

These effects could result in two consequences simultaneously. On one hand, the agglomeration of polyelectrolyte complexes, PNR-DNA’s led to the appearance of the sites with thinner PNR coating. The agglomeration provided more availability of polymer for electron exchange and, consequently, the growth of (Ia/Ic) peak pair currents. At the same time, when the “tightening” process took place, the bridges between monomer units could be destroyed, as well as the electron transfer could be complicated by the steric factors and the response of the (IIa/IIc) peaks pair could decrease.

**Figure 11:**
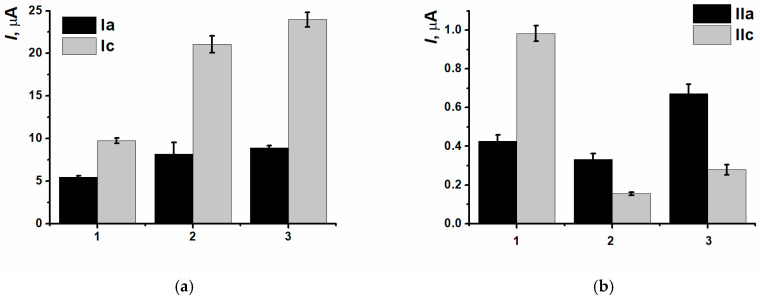
Peak currents changing for (1) SPCE/PNR_REL_, (2) SPCE/PNR_REL_/DNAss, (3) SPCE/PNR_REL_/PSS. (**a**) peaks pair (Ia/Ic), (**b**) peaks pair (IIa/IIc).

To establish the DNAss influence on the sensor response, there were some experiments with PSS polyanion were carried out. Although the peak current values were different, the trend of their change was the same (Figure 11), suggesting the DNAss influence was defined not only by the negative charge density of the modifying layer but the biopolymer nature itself.

**Figure 12:**
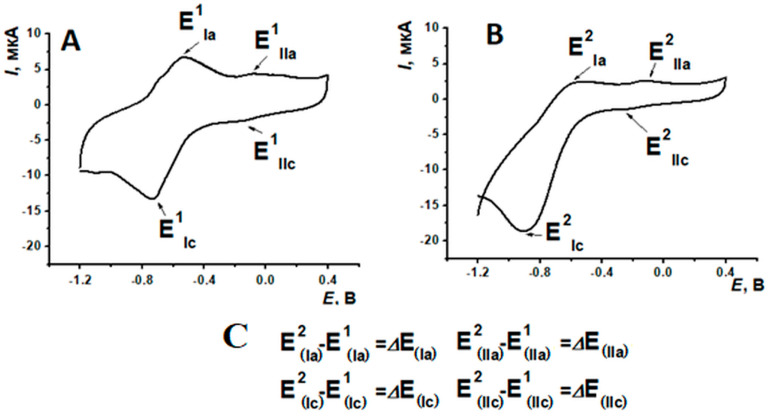
PNR response before (**A**) and after (**B**) DNAss implementation; (**C**) experiment results processing scheme.

As DNA inclusion into the coating has led to nanocomposite peak currents changing (Table 4), as well as the potentials shift, the next scheme of experimental data processing was suggested (Figure 12).

While the changes in redox currents value recorded on the voltammograms after the DNA non-conductive molecules immobilization are expected, the sufficient shifts in potentials are less common. Usually, in case of insufficient current changes obtained in cyclic voltammetry, one can turn to another electrochemical method such as square wave voltammetry, differential pulse voltammetry, or electrochemical impedance spectroscopy. The transfer from cyclic voltammetry to square wave or differential pulse voltammetry sometimes cannot meet the specific challenges related to the measurements [29]. In this case, the use of electrochemical impedance spectroscopy can be more sensitive to the DNA damage or specific DNA interactions with low-molecular compounds in the biorecognizing layer [29,30].

As can be seen from Table 4, the incubation in PSS solution had no significant influence on peak potentials, whereas DNAss inclusion into the coating has led to the potential shift towards the cathodic area. It is noteworthy that the maximal change in peak position was observed for the (Ic) peak potential, being about 200 mV. It was suggested to use ∆E_(Ic)_ and ∆E_(IIc)_ for graphical presentation of the analytical response changes obtained according to the protocol mentioned above.

When the incubation time in the DNAss solution was varied, there were no significant changes in peak currents after 10 min for the first pair (Ia/Ic) observed (Figure S7). At the same time, the maximal changes in the reduction peak potentials were recorded for the coatings incubated in DNA solution for 10 min (Table S2). When the DNA concentration increased, there was peak current monotonous growing for the pair (Ia/Ic), whereas for (IIa/IIc) the dependency changed irregularly (Figure S8). The same behavior was observed for the peak potentials of (Ic) и (IIc) (Figure S9). For the first peak, gradual potential shift increased with the following stabilization after 1 mg/mL. This could indicate the modification of coating saturation with DNA molecules. ∆E_(IIc)_ was predominately varied for 20–30 mV with maximal shifting on 88 mV after incubation in 1 mg/mL DNAss solution. Considering all the facts mentioned, the following experiments were carried out with 10 min incubation in 1 mg/mL DNAss solution.

**Figure 13:**
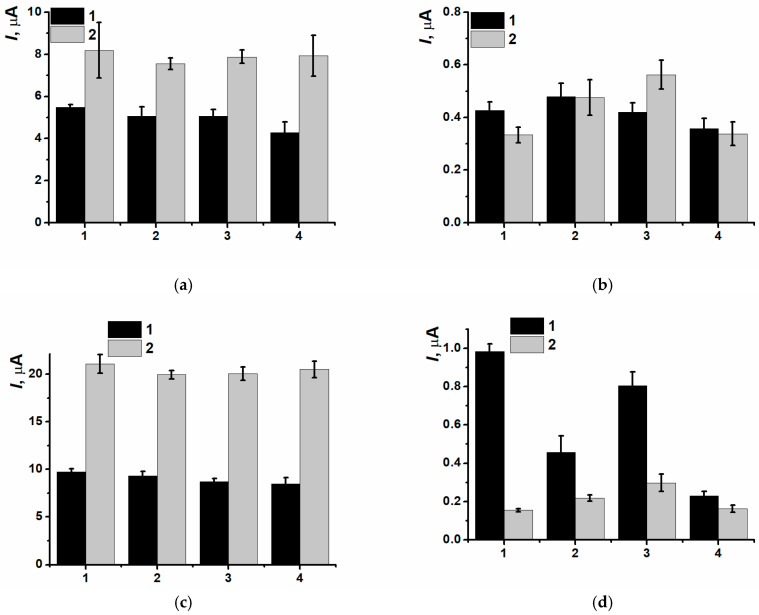
(**a**,**b**) Oxidation and (**c**,**d**) reduction peak currents dependencies on different type of CNM inclusion before (1) and after (2) DNAss drop casting onto the composite surface, (1) SPCE/PNR_REL_, (2) SPCE/PNR_REL_(CB_250G_), (3) SPCE/PNR_REL_(CB_350G_), (4) SPCE/PNR_REL_(MWCNTf).

CNM presence in the PNR film had not affected the first peaks pair (Ia/Ic) changes when DNAss was included in the coating (Figure 13).

**Figure 14:**
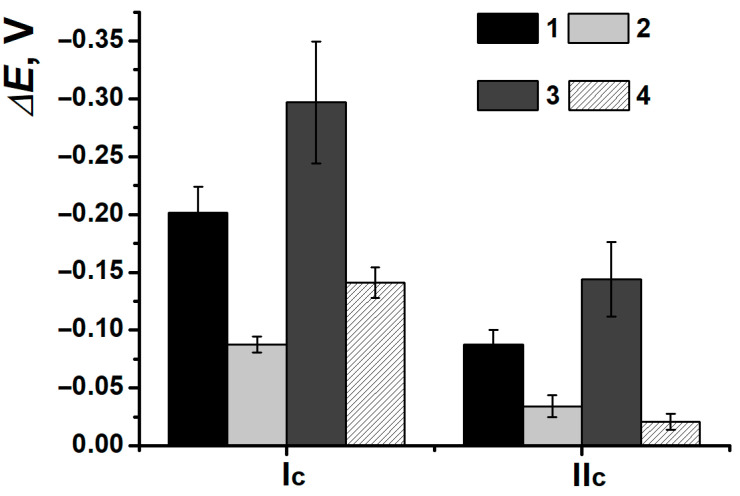
Reduction peak potentials shift for the composites (1) SPCE/PNR_REL_, (2) SPCE/PNR_REL_(CB_250G_), (3) SPCE/PNR_REL_(CB_350G_), (4) SPCE/PNR_REL_(MWCNTf) after DNAss implementation.

The most informative approach appeared to use the pair (IIa/IIc) currents and the shift in reduction peak potential after its incubation in DNAss solution (Figure 14).

As the redox processes of NR directly depended on the rate of equilibrium reached between oxidized and reduced forms in the film, and so, the rate of counter ions transfer processes, even weak changing of peaks potentials after the contact with DNAss solution for the coatings SPCE/PNR(CB_250G_) and SPCE/PNR(MWCNTf) could indicate the absence of steric and diffusional counter ions transfer complications. Therefore, the presence of MWCNTf and CB_250G_ in the composite coating decreased the efficiency of biopolymer immobilization, whereas the inclusion of CB_350G_ promoted closer contact of DNAss with the polymeric units. That was the reason to use composite coating based on PNR_REL_ electrodeposited in presence of CB_350G_ for the following experiments.

### 3.5. Detection of DNA Damage

Various endogenous or exogenous agents can influence DNA in cells, which results in chemical and spatial changes in its structure. This definitely affects human health [31]. DNA damages directly influence on the cell vitality, gene information safety, organism activity and mutations occurrence with the following pathologic situations development. In all cases, the interaction between positively charged poly(Neutral red) in the nanocomposite content and negatively charged DNA (native, thermally denatured, or oxidized) occurred due to the electrostatic forces. When DNA had been thermally damaged, there was the destruction of hydrogen or other types of binding between complementary chains of DNA with their following partial dehybridization. The investigation of thermal denaturation of DNA allows us to study physico-chemical properties of DNA more carefully, which is important in biotechnology, for example, for PCR tests. Similarly, oxidative damage of DNA with ROS leads to human immune system suppression, aging and oncology or neurodegenerative decease development. Therefore, the development of registration approaches for such kinds of DNA damage can become a key to a deeper understanding of molecular mechanisms and novel diagnostic methods.

Three types of oxidative agents were used to assess the possibility of DNA oxidative damage discrimination, such as the Fenton reagent, mixture of Cu^2+^ and H_2_O_2_, and H_2_O_2_ itself. The interaction of DNA with the components of the oxidizing mixture led to the hydroxide radical formation [32] which provided DNA double stranded structure to be damaged [33,34]. DNA damage changes the flexibility, spatial structure, and the charge distribution in the DNA helix that can be detected with electrochemical methods.

**Figure 15:**
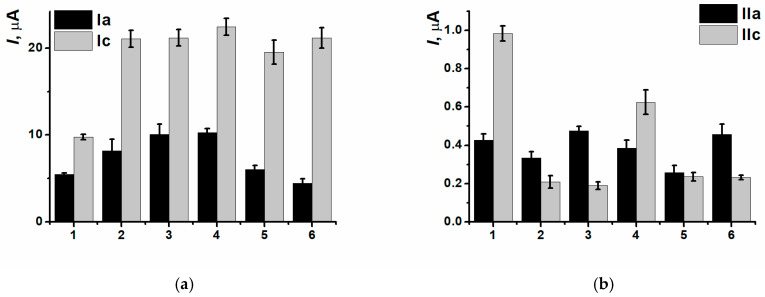
DNAss damage discrimination on (1) SPCE/PNR_REL_, (2) SPCE/PNR_REL_/DNAss, (3) SPCE/PNR_REL_/DNAden, (4) SPCE/PNR_REL_/DNAox(Cu), (5) SPCE/PNR_REL_/DNAox(F), (6) SPCE/PNR_REL_/DNAox(H_2_O_2_) with (Ia/Ic) (**a**) and (IIa/IIc) (**b**) peak currents.

**Figure 16:**
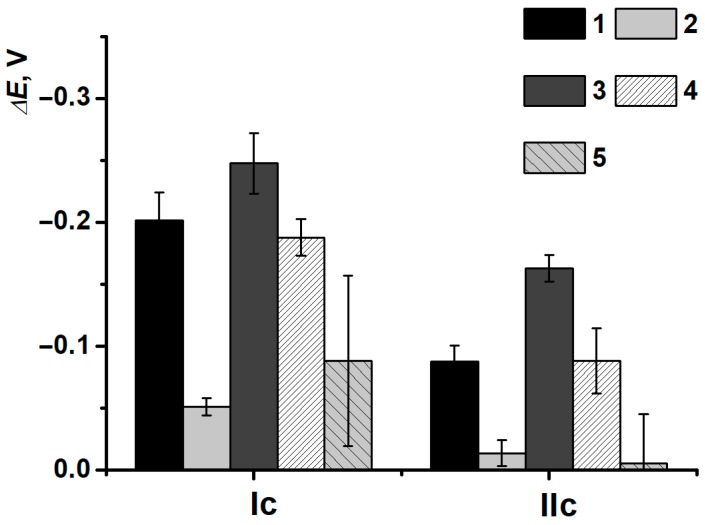
DNAss damage discrimination on (1) SPCE/PNR_REL_/DNAss, (2) SPCE/PNR_REL_/DNAden, (3) SPCE/PNR_REL_/DNAox(H_2_O_2_), (4) SPCE/PNR_REL_/DNAox(Cu), (5) SPCE/PNR_REL_/DNAox(F) with reduction peak potentials.

For PNR_REL_ peak currents of (Ia), the only discrimination between damages caused by Fenton reagent (DNAox(F)) and H_2_O_2_ (DNAox(H_2_O_2_)) was possible (Figure 15), as the coatings with DNAden and DNAox(Cu) gave a similar response. The second pair of peaks in this case was more informative. The growth of (IIa/IIc) currents was provided by the increasing availability of nitrogen in inter-unit bridges for the electron exchange, as double-stranded DNAss structure damage interfered with the processes of its partial intercalation by the dye. The difference between the changes in peak currents when various damaging agents were used could be related to the interfering influence of metal cations formed as a result of ROS presence and/or their complexes with DNAss formation. The appropriate changes in peak potentials depending on DNA damage are presented in Figure 16.

In this case, only the thermal denaturation and H_2_O_2_ oxidation of DNAss could be discriminated. The determination of oxidative damage in the presence of Cu^2+^ and H_2_O_2_ mixture was possible only with the (IIc) peak current, whereas for the damage with the Fenton reagent, the high deviation of the results should be noticed, which could be provided by a great amount of uncontrolled side reactions and processes.

**Figure 17:**
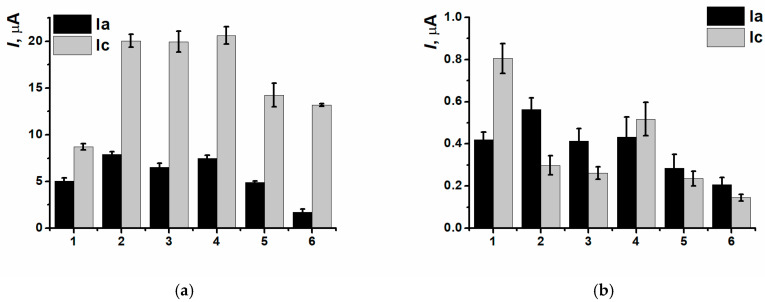
DNAss damage discrimination on (1) SPCE/PNR_REL_(CB_350G_), (2) SPCE/PNR_REL_(CB_350G_)/DNAss, (3) SPCE/PNR_REL_(CB_350G_)/DNAden, (4) SPCE/PNR_REL_(CB_350G_)/DNAox(Cu), (5) SPCE/PNR_REL_(CB_350G_)/DNAox(F), (6) SPCE/PNR_REL_(CB_350G_)/DNAox(H_2_O_2_) with (Ia/Ic) (**a**) and (IIa/IIc) (**b**) peak currents.

After the interaction between nanocomposite polymeric coating PNR_REL_(CB_350G_) and thermally denatured or DNAss oxidized by Cu^2+^ and H_2_O_2_ mixture, there were no any significant changes in (Ia/Ic) peak currents observed, so, the discrimination of all the types of biomolecule damage with peak currents stayed possible only for the pair (IIa/IIc) (Figure 17).

**Figure 18:**
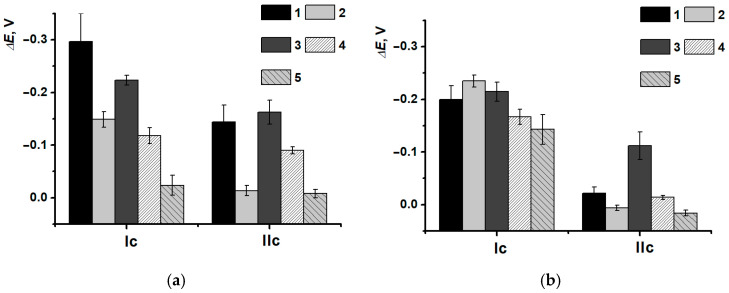
(**a**) DNAss and (**b**) DNAch damage discrimination on (1) SPCE/PNR_REL_(CB_350G_)/DNA, (2) SPCE/PNR_REL_(CB_350G_)/DNAden, (3) SPCE/PNR_REL_(CB_350G_)/DNAox(Cu), (4) SPCE/PNR_REL_(CB_350G_)/DNAox(F), (5) SPCE/PNR_REL_(CB_350G_)/DNAox(H_2_O_2_) with reduction peak potentials changes.

However, another behavior of (IIa/IIc) peak currents and reduction peak potentials was observed in the presence of CB. Due to a great shift in potentials for SPCE/PNR_REL_(CB_350G_) when DNAss was included, all the damage discriminations became possible (Figure 18a). It is noteworthy that the deviation of the determination decreased, i.e., CB_350G_ inclusion allowed to stabilize the coating and obtain more reproducible polymeric film morphology, as DNAss had the predominant influence on the outer coating layer without entering the composite.

DNA damage degree is considerably provided by the biopolymer conformation, average molecular weight of nucleotides sequences and the presence of chain sites enriched with guanine fragments. The DNA biosensor assembling protocol developed was tested for high-molecular-weight DNAch.

After the incubation in high-molecular-weight DNAch, the shift in peak potentials was less obvious (Figure 18b), which could indirectly indicate the formation of denser polyelectrolyte complexes. The latter ones could slow down the processes of redox equilibrium reaching in the polymeric film. Potential shift in (Ic) turned out to be more sensitive towards damaged DNAch fragments (all damage types could be detected), whereas the reduction peak potential shift in (IIc) was constant (except for the damage with H_2_O_2_ solution).

**Figure 19:**
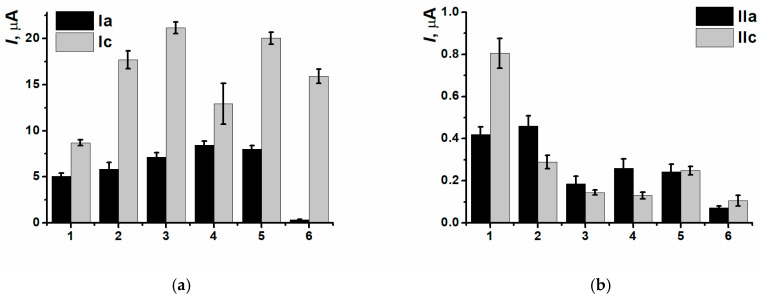
DNAch damage discrimination on (1) SPCE/PNR_REL_(CB_350G_), (2) SPCE/PNR_REL_(CB_350G_)/DNAch, (3) SPCE/PNR_REL_(CB_350G_)/DNAden, (4) SPCE/PNR_REL_(CB_350G_)/DNAox(Cu), (5) SPCE/PNR_REL_(CB_350G_)/DNAox(F), (6) SPCE/PNR_REL_(CB_350G_)/DNAox(H_2_O_2_) with (Ia/Ic) (**a**) and (IIa/IIc) (**b**) peak currents.

At the same time, the combination of high molecular weighted DNAch and composite PNR_REL_(CB_350G_) allowed to establish all the types of damages for both pairs of peak currents (Figure 19) definitely.

The discrimination of Cu^2+^ and H_2_O_2_ mixture damage became real due to several reasons related to DNAch and DNAss structures. Firstly, DNAss is presented with simple conformations (mostly short linear chains), whereas DNAch is able to form more complicated and compact structures, such as a superhelix, which makes steric complications play a role in specific interactions with metal ions. For this reason, Cu^2+^ took part only in ROS formation and did not form complexes with DNAch. Secondly, DNAch is rich with guanine-cytosine fragments (G-quadruplexes), which are more subjected to oxidative stress.

As the screen-printed electrodes are a low-cost platform for the sensor and biosensor assembly, the reagents expense is minimal, and the scaling of the technology is simple; there is no need to perform a sensor for multiple uses. Disposable sensors allow for the prevention of the interfering effects or “memory effect” when transferring the sensor from one sample to another. Single-use is the main promising feature of the sensors developed for food control and medical diagnostics.

The sensors were stored in the dry conditions at 4 °C under Eppendorf tubes (in the fridge. The standard deviation of the sensor response was 5.3% when using it just after the assembly protocol realization. After 7 days of storage, the value had a 12.4% deviation, and after 2 weeks, the value deviated by 19.8% which makes the determination with the sensors after 14 days of storage insufficient.

As DNA biosensors allowed to imitate the conditions of cell oxidative stress and assess damage degree in presence of the antioxidants [35,36], it was interesting to carry out the tests of the sensor developed based on DNAch and composite/PNR_REL_(CB_350G_) for general antioxidant activity establishment. Purchased in the local market, green tea extracts of three trademarks, coffee, white wine, cocoa, fruit-based drink, and Effervescent Vitamin C were used as real samples. The first pair (Ia/Ic) peak currents were decided to be taken for analytical response for processing simplicity.

**Figure 20:**
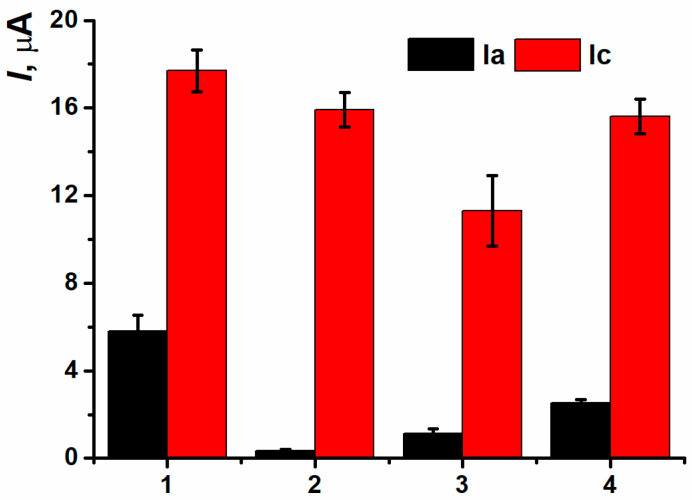
The assessment of tea antioxidant protective effect with (Ia/Ic) peak currents on SPCE/PNR_REL_(CB_350G_)/DNAch, (1) native DNA, (2) H_2_O_2_ oxidized DNA, (3) tea 1, (4) tea 2.

The assessment of antioxidant protective effect was carried out in two ways. In the first case, 100 µL of the tea sample studied and 1.32 µL H_2_O_2_ solution were consequently added to the DNAch solution (tea 1). In the second case, a previously prepared mixture of 100 µL of green tea and 1.32 µL H_2_O_2_ was added to DNAch (tea 2). The values of (Ia) peak currents were between those for native and oxidized DNAch (Figure 20) which indicated the protective antioxidant properties of green tea. The second approach to antioxidant protective effect assessment with previous mixing of the oxidizing agent and antioxidant sample turned out to be more reproducible, so it was chosen for the following experiments.

**Figure 21:**
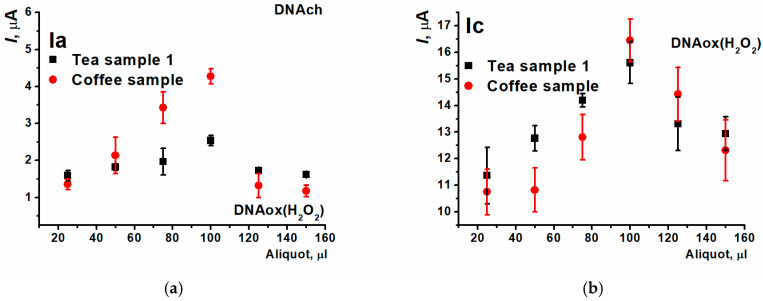
The dependence of beverage antioxidant protective effect on their aliquot on SPCE/PNR_REL_(CB_350G_)/DNAch with (**a**) (Ia) and (**b**) (Ic) peak currents.

Peak currents of the (Ia/Ic) pair were increasing with the volume of the beverage sample added (Figure 21). However, only the oxidation peak current could allow to assess the antioxidant protective effect because all the results obtained for different coffee and tea aliquots were in the range between the currents of native and oxidized DNAch. Maximal change in the currents compared to the coating based on oxidized DNAch was recorded when the aliquot being equal 100 µL. The following decrease in the currents recorded with higher aliquots could be provided by prooxidant effect.

**Figure 22:**
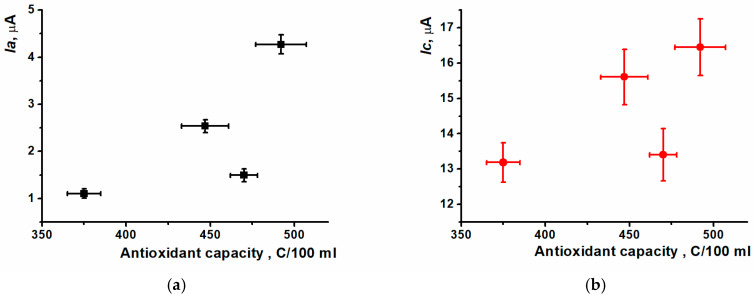
Comparative characteristics of antioxidant capacity and (Ia/Ic) peak currents for tea and coffee samples. (**a**) oxidation and (**b**) reduction peak currents.

For the most samples tested, the simultaneous changing of oxidation peak currents and antioxidant capacity was observed (Figure 22).

There was no complete correlation between oxidation peak currents for all beverage samples and the results obtained with an independent assessment approach (coulometric titration) for integral antioxidant capacity with bromine (Table 5). It could be explained by the presence of artificial additives in the tea sample 3 affecting the antioxidant capacity value. The results obtained for white wine, cocoa, fruit-based drink, and effervescent Vitamin C are presented in Table 5. The samples of white vine and fruit-based drink had rather low levels of antioxidant capacity; the deviation of the correlation for the last sample could be attributed to the presence of flavorings and artificial additives. The cocoa sample demonstrated a medium level of antioxidant capacity. The effervescent Vitamin C showed the closest value to the response of tea samples. Anyway, the DNA biosensor developed can find its application in control of food products quality.

## 4. Conclusions

In the manuscript presented the electropolymerization of Neutral red phenazine dye from deep eutectic solvent reline on screen-printed carbon electrodes was described and compared to convenient one in aqueous medium. The difference in electrochemical characteristics of the polymer coatings was shown. Also, poly(Neutral red) was used with various nanomaterials to obtain a range of novel nanocomposites from a deep eutectic solvent. Combining several modification steps into a single one allowed us to simplify and speed up the DNA biosensor assembly protocol. The inclusion of various carbon nanomaterials affected the coating morphology and the electrochemical characteristics of the nanocomposites obtained. The most efficient assembly of nanocomposite was possible with CB_350G_ using compared to CB_250G_ and MWCNTf. DNA implementation into the modifying layer has changed the currents recorded and led to cathodic potential shift. The DNA source and its molecular weight had a significant influence on electron transfer processes inside the modifying layer. It should be noted that the DNA immobilization protocol, as well as its loading and incubation time, were optimized to provide the maximal analytical response of the DNA biosensor developed. The biosensor response was subjected to different types of DNA damage, including thermal denaturation and chemical oxidation with H_2_O_2_ only, Fenton reagent, or a mixture of Cu^2+^ and H_2_O_2_. The DNA biosensor developed was used to assess the antioxidant capacity of tea and coffee samples. The validity of voltammetric approach proposed was confirmed by coulometric titration with electrogenerated bromine.

Successful application of the DNA biosensor developed in real beverage samples allows us to suggest the possibility of its further use for monitoring of food quality and antioxidant additives. Future prospects of the disposable DNA biosensors based on the SPCE modified with electropolymerized materials from DES media are associated with the extension of the range of monomers used and the transfer from the binary DES mixtures to the tertiary ones. The stability improvement depends on the combination of natural bioreceptor with stabilizing compounds in modifying layer. Novel protocols of bioreceptor implementation are another challenge to be solved in the future.

## Figures and Tables

**Table 1:** Linear regression equations of bilogarithmic log*I*_p_–log*v* dependencies for peaks pair (Ia/Ic) for SPCE/PNR_PB_ and SPCE/PNR_REL_.

log*I_p_* = a + b × log*v*	
SPCE/PNR_PB_	log(*I*_(Ia)_, µA) = −(0.42 ± 0.059) + (0.62 ± 0.032) × log(ν, mV/s) R^2^ = 0.965
	log(*I*_(Ic)_, µA) = (0.14 ± 0.027) + (0.49 ± 0.015) × log(ν, mV/s) R^2^ = 0.995
SPCE/PNR_REL_	log(*I*_(Ia)_, µA) = −(0.49 ± 0.029) + (0.56 ± 0.031) × log(ν, mV/s) R^2^ = 0.992
	log(*I*_(Ic)_, µA) = (0.15 ± 0.006) + (0.50 ± 0.004) × log(ν, mV/s) R^2^ = 0.997

**Table 2:** Linear regression equations of bilogarithmic log*I*_p_–log*v* dependencies for peaks pair (IIa/IIc) for SPCE/PNR_PB_ and SPCE/PNR_REL_.

log*I_p_* = a + b × log*v*	
SPCE/PNR_PB_	log(I(IIa), µA) = −(1.13 ± 0.014) + (0.68 ± 0.026) × log(ν, mV/s) R^2^ = 0.991
	log(I(IIc), µA) = −(1.29 ± 0.014) + (0.73 ± 0.027) × log(ν, mV/s) R^2^ = 0.987
SPCE/PNR_REL_	log(I(IIa), µA) = −(1.88 ± 0.030) + (0.91 ± 0.062) × log(ν, mV/s) R^2^ = 0.979
	log(I(IIc), µA) = −(2.23 ± 0.030) + (1.01 ± 0.059) × log(ν, mV/s) R^2^ = 0.988

**Table 3:** The slopes of d*E_m_*/dpH of linear ranges of equilibrium peak potential dependencies on pH value.

*E_m_* = a + b × pH					
		pH Range	*a* ± Δa	*b* ± Δb	R^2^
SPCE/PNR_PB_	(Ia/Ic)	3.0–5.0	−0.476 ± 0.015	−0.056 ± 0.003	0.99
		5.0–7.0	−0.771 ± 0.029	0.0036 ± 0.005	0.95
		7.0–9.0	−0.433 ± 0.078	−0.045 ± 0.011	0.90
	(IIa/IIc)	3.0–9.0	0.135 ± 0.02	−0.048 ± 0.003	0.97
SPCE/PNR_REL_	(Ia/Ic)	3.0–5.0	−0.448 ± 0.051	−0.033 ± 0.012	0.87
		5.0–7.0	−0.532 ± 0.001	−0.015 ± 0.001	0.99
		7.0–9.0	−0.378 ± 0.088	−0.037 ± 0.012	0.89
	(IIa/IIc)	3.0–9.0	0.246 ± 0.019	−0.054 ± 0.003	0.98

**Table 4:** Peak pairs potentials of modifying coating after its stabilization and incubation in DNAss or PSS solution.

Layer content	*E* ^1^_(Ia)_	*E* ^1^_(Ic)_	*E* ^1^_(IIa)_	*E* ^1^_(IIc)_
SPCE/PNR_REL_	−0.548 ± 0.016	−0.925 ± 0.020	−0.108 ± 0.009	−0.200 ± 0.013
	*E* ^2^_(Ia)_	*E* ^2^_(Ic)_	*E* ^2^_(IIa)_	*E* ^2^_(IIc)_
SPCE/PNR_REL_/DNAss	−0.503 ± 0.007	−0.724 ± 0.014	−0.069 ± 0.003	−0.112 ± 0.009
SPCE/PNR_REL_/PSS	−0.547 ± 0.010	−0.936 ± 0.015	−0.134 ± 0.007	−0.196 ± 0.008

**Table 5:** Comparison of DNA biosensor (Ia/Ic) peak currents recorded and antioxidant capacity of beverage samples.

	Ia, µA	Ic, µA	AC, C/100 mL
Tea sample 1	2.54 ± 0.14	15.6 ± 0.79	447 ± 14, Sr = 0.026
Tea sample 2	1.10 ± 0.10	13.2 ± 0.55	375 ± 10, Sr = 0.011
Tea sample 3	1.49 ± 0.14	13.4 ± 0.74	470 ± 8, Sr = 0.013
Coffee sample	4.27 ± 0.20	16.5 ± 0.81	492 ± 15, Sr = 0.025
White wine sample	0.39 ± 0.25	5.85 ± 2.6	20 ± 1, Sr = 0.046
Cocoa sample	1.03 ± 0.20	10.8 ± 0.51	207 ± 7, Sr = 0.037
Effervescent Vitamin C sample	1.17 ± 0.03	4.62 ± 0.77	374 ± 4, Sr = 0.023
Fruit-based drink sample	0.83 ± 0.17	13.06 ± 1.5	43 ± 1, Sr = 0.013

## Data Availability

The original contributions presented in this study are included in the article/Supplementary Materials. Further inquiries can be directed to the corresponding author.

## Supplementary Materials

The following supporting information can be downloaded at https://www.mdpi.com/article/10.3390/bios15110735/s1, Figure S1. Cyclic voltammograms obtained for SPCE/PNR_PB_ (A) and SPCE/PNR_REL_ (B) in 0.04 M BRB at various pH; Figure S2. Equilibrium potential of peaks pair (Ia/Ic) (A) and (IIa/IIc) (B) dependency on 0.1 M PB pH value for SPCE/PNR_PB_ (A) and SPCE/PNR_REL_; Figure S3. Equilibrium potential of peak pair (Ia/Ic) (A) and (IIa/IIc) (B) dependency on 0.04 M BRB pH value for SPCE/PNR_PB_ (A) and SPCE/PNR_REL_; Figure S4. Voltammograms of NR polymerization in reline onto supporting CNM layer: CB_250G_ (A), CB_350G_ (B) and MWCNTf (C). Red is for the 1st cycle; Figure S5. Cyclic voltammograms of PNR_REL_ on the supporting CNM layer: (1) SPCE/PNR_REL_; (2) SPCE/MWCNTf/PNR_REL_; (3) SPCE/CB_250G_/PNR_REL_; (4) SPCE/CB_350G_/PNR_REL_; Figure S6. Peak currents of pairs (Ia/Ic) (A) and (IIa/IIc) (B) of PNR_REL_ on the supporting CNM layer: (1) SPCE/PNR_REL_; (2) SPCE/CB_250G_/PNR_REL_; (3) SPCE/CB_350G_/PNR_REL_, (4) SPCE/MWCNTf/PNR_REL_; Figure S7. Peak currents (Ia/Ic) (A) and (IIa/IIc) (B) comparison for the coatings with different time of SPCE/PNR_REL_ incubation in DNAss solution: (1) no DNA, (2) 5 min, (3) 10 min, (4) 15 min, (5) 20 min, (6) DNA drying; Figure S8. Comparison of peak currents for Ia/Ic (A) and IIa/IIc (B), obtained for SPCE/PNR_REL_ incubated in DNAss solution with different concentrations: (1) no DNA, (2) 100 ng/mL, (3) 1 µg/mL, (4) 10 µg/mL, (5) 100 µg/mL, (6) 1 mg/mL, (7) 2 mg/mL; Figure S9. Cathodic potential shift for (Ic) and (IIc) peaks on SPCE/PNR_REL_/DNAss depending on DNAss concentration; Table S1. Slopes of dependency of peaks’ formal potential on pH in 0.04 M BRB; Table S2. (Ic) and (IIc) peak potential shifts for different incubation times in DNAss solution.
