# Supplementary material for: Novel Nanocomposites of Carbon Nanomaterials and Poly(Neutral Red) Electropolymerized from Reline for DNA Damage Detection and Beverage Antioxidant Influence Assessment
Source: Biosensors (Basel). 2025 Nov 3;15(11):735. doi: 10.3390/bios15110735 (PMC12650678; doi:10.3390/bios15110735)
Supplement: Supplementary file 1 [file biosensors-15-00735-s001.zip › biosensors-3842328-supplementary.pdf]

Electronic Supporting Information

to the article of Anastasia Malanina, Rufiia Derbisheva, Tatiana Krasnova, Rezeda Shamagsumova, Vladimir Evtugyn, Alexey Ivanov, and Anna Porfireva

Novel Nanocomposites of Carbon Nanomaterials and Poly(Neutral Red) Electropolymerized from Reline for DNA Damage Detection and Beverage Antioxidant Influence Assessment

A) B)

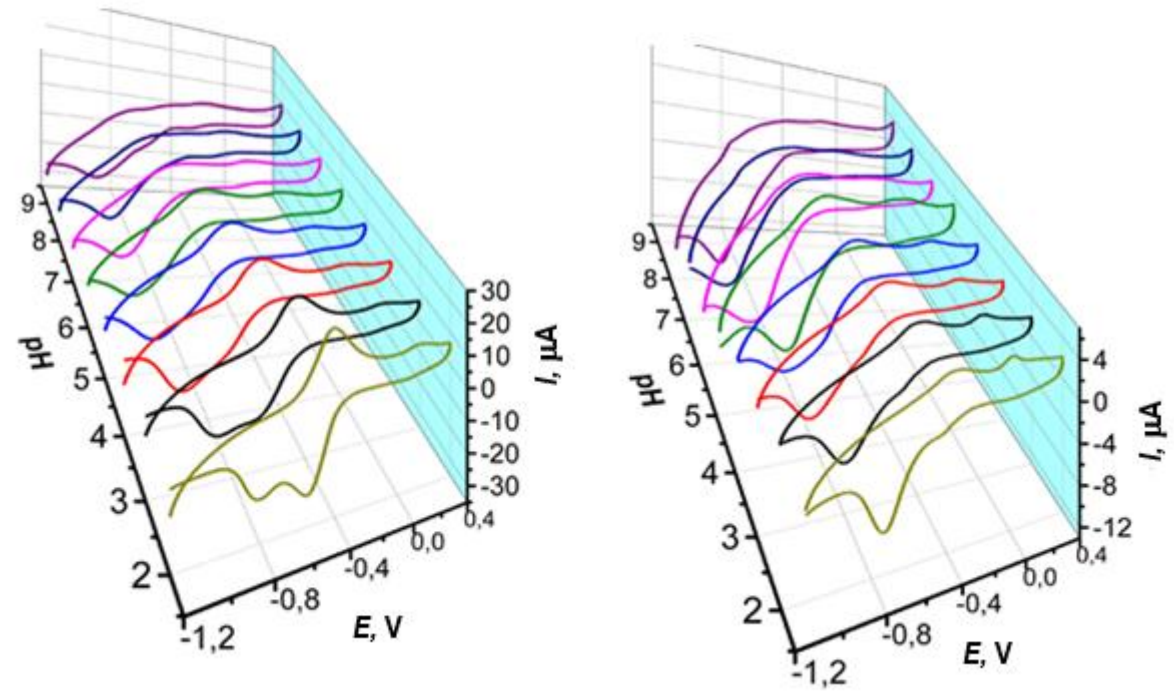

Figure S1. Cyclic voltammograms obtained for SPCE/PNR$_{PB}$ (A) and SPCE/PNR$_{REL}$ (B) in 0.04 M BRB at various pH

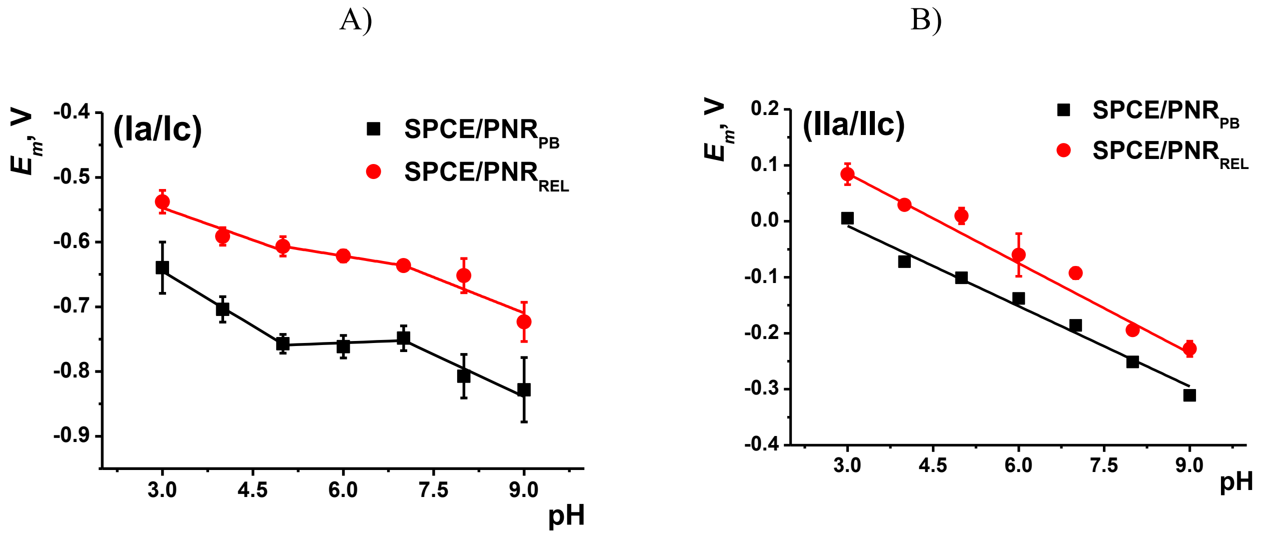

Figure S2. Equilibrium potential of peaks pair (Ia/Ic) (A) and (IIa/IIc) (B) dependency on 0.1 M PB pH value for SPCE/PNR$_{PB}$ (A) and SPCE/PNR$_{REL}$

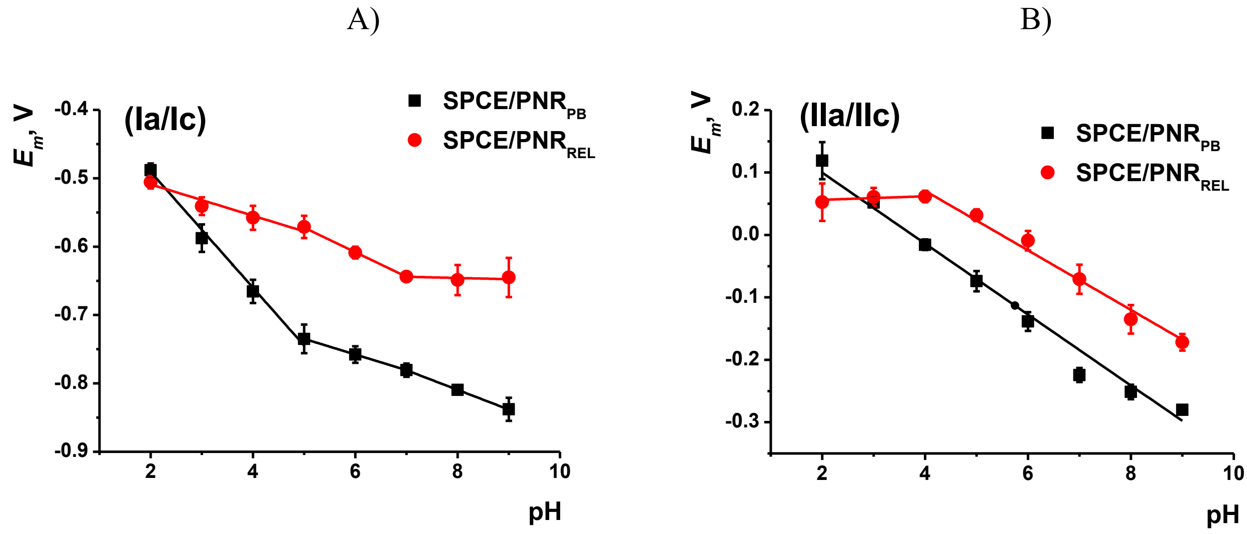

Figure S3. Equilibrium potential of peak pair (Ia/Ic) (A) and (IIa/IIc) (B) dependency on 0.04 M BRB pH value for SPCE/PNR$_{PB}$ (A) and SPCE/PNR$_{REL}$

Table S1. Slopes of dependency of peaks formal potential on pH in 0.04 M BRB

$E_m = a + b \times pH$					
		pH range	$a \pm \Delta a$	$b \pm \Delta b$	R^2
$SPCE/PNR_{PB}$	(Ia/Ic)	2.0-5.0	-0.321±0.012	-0.085±0.004	0.99
		5.0-7.0	-0.620±0.001	-0.022±0.0001	0.99
		7.0-9.0	-0.579±0.001	-0.029±0.0001	0.99
	(IIa/IIc)	2.0-9.0	0.14±0.018	-0.057±0.003	0.98
$SPCE/PNR_{REL}$	(Ia/Ic)	2.0-5.0	-0.463±0.011	-0.023±0.004	0.93
		5.0-7.0	-0.393±0.004	-0.036±0.001	0.99
		7.0-9.0	-0.631±0.013	-0.002±0.002	0.89
	(IIa/IIc)	2.0-4.0	0.051±0.008	0.003±0.002	0.87
		4.0-9.0	0.261±0.016	-0.048±0.003	0.98

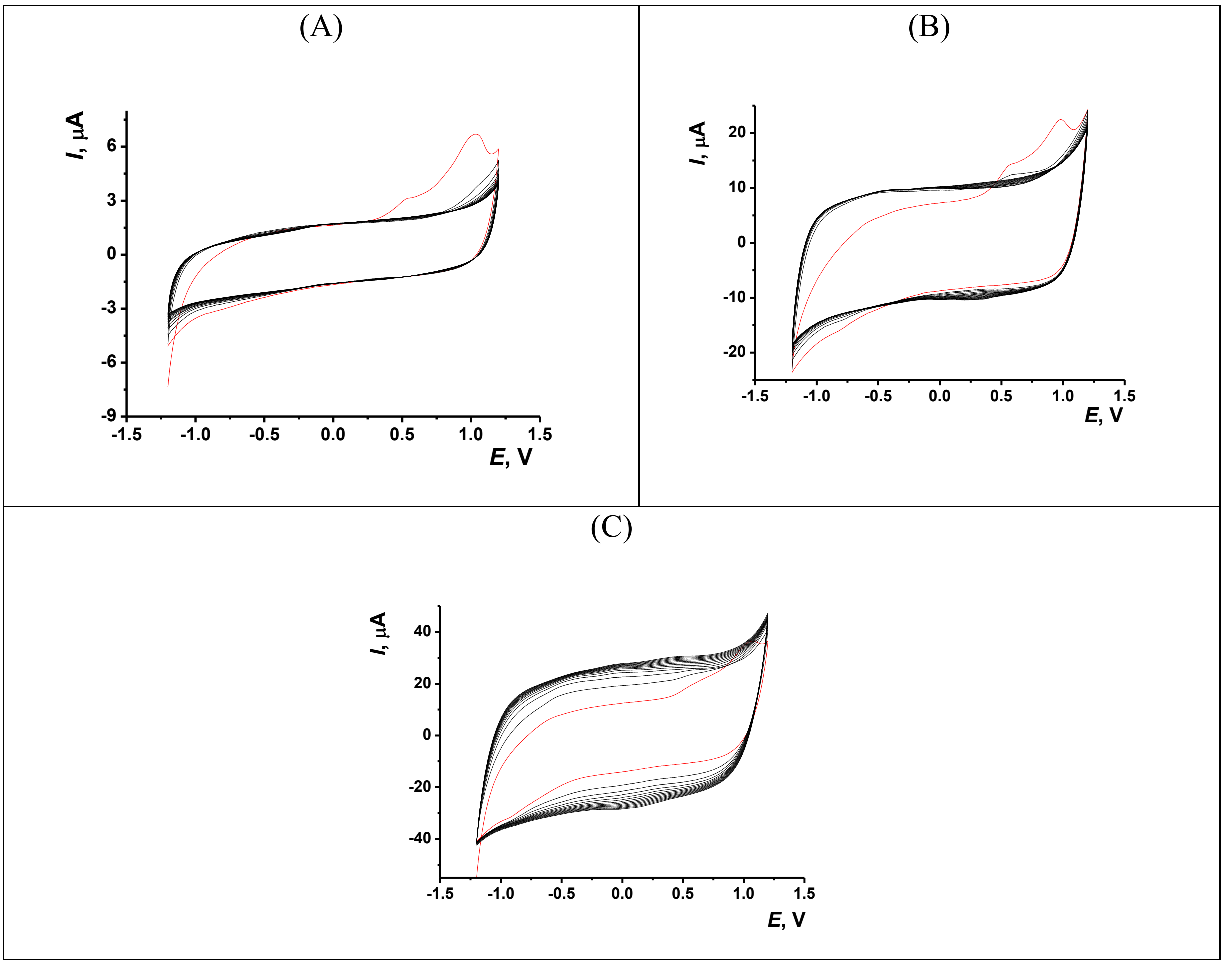

Figure S4. Voltammograms of NR polymerization in reline onto supporting CNM layer: CB_{250G} (A), CB_{350G} (B) and MWCNTf (C). Red is for the 1st cycle

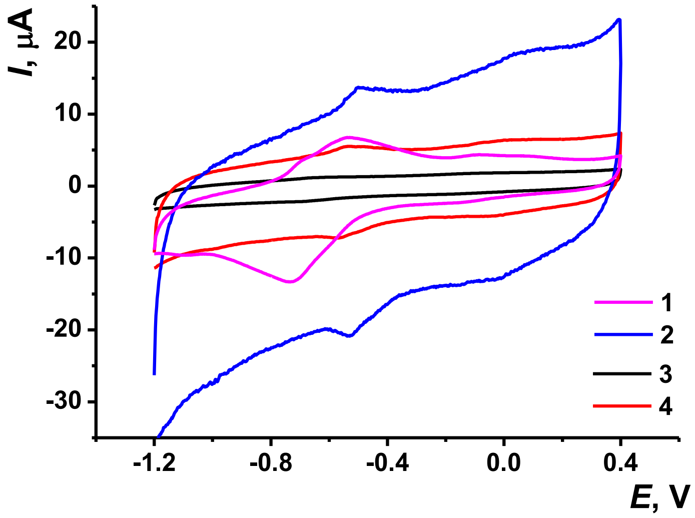

Figure S5. Cyclic voltammograms of PNR_{REL} on the supporting CNM layer: (1) SPCE/PNR_{REL}; (2) SPCE/MWCNTf/PNR_{REL}; (3) SPCE/CB_{250G}/PNR_{REL}; (4) SPCE/CB_{350G}/PNR_{REL}

A) B)

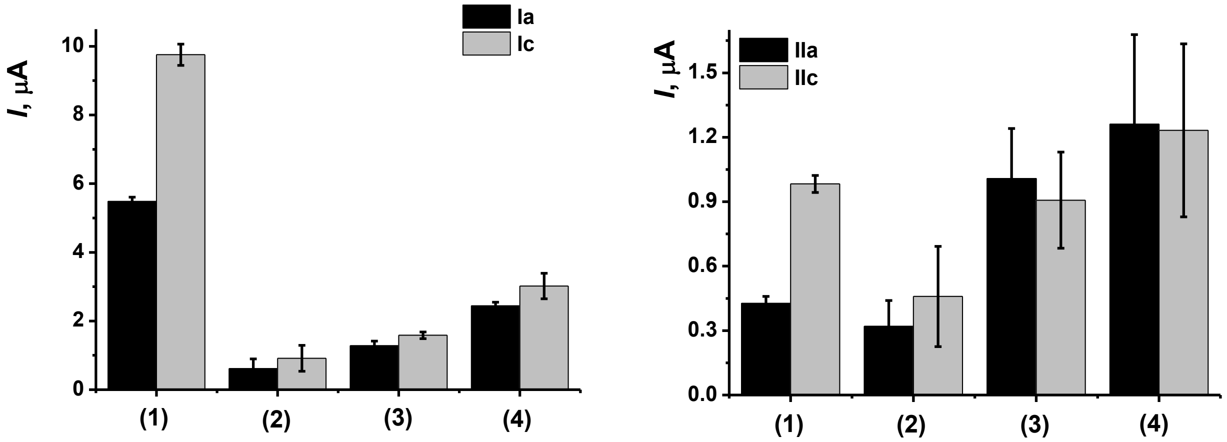

Figure S6. Peak currents of pairs (Ia/Ic) (A) and (IIa/IIc) (B) of PNR_{REL} on the supporting CNM layer: (1) SPCE/PNR_{REL}; (2) SPCE/CB_{250G}/PNR_{REL}; (3) SPCE/CB_{350G}/PNR_{REL}, (4) SPCE/MWCNTf/PNR_{REL}

A) B)

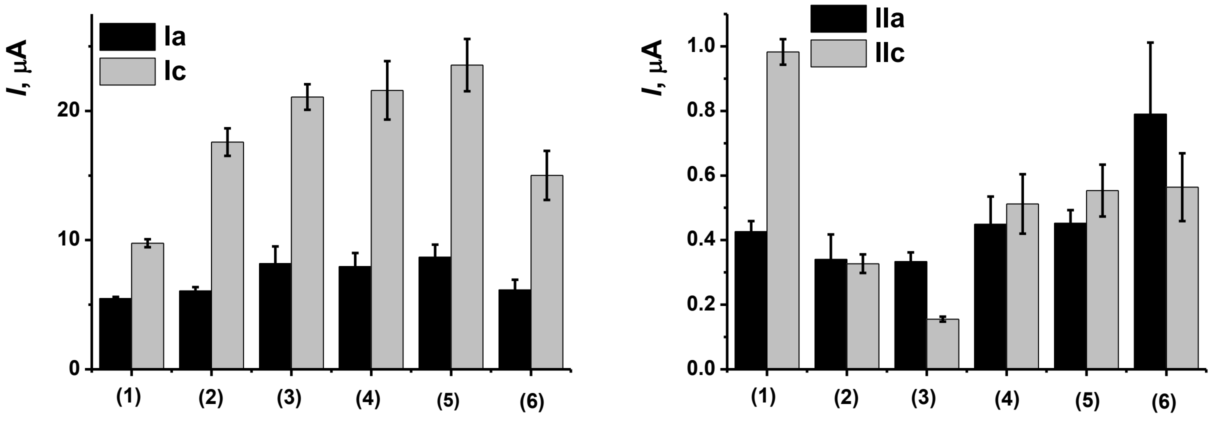

Figure S7. Peak currents (Ia/Ic) (A) and (IIa/IIc) (B) comparison for the coatings with different time of SPCE/PNR$_{REL}$ incubation in DNAss solution: (1) no DNA, (2) 5 minutes, (3) 10 minutes, (4) 15 minutes, (5) 20 minutes, (6) DNA drying

Table S2. (Ic) and (IIc) peak potential shifts for different incubation time in DNAss solution

Incubation time	$\Delta E_{(Ic)}$	$\Delta E_{(IIc)}$
5 min	-0.103±0.008	-0.018±0.014
10 min	-0.202±0.023	-0.088±0.013
15 min	-0.104±0.032	-0.020±0.012
20 min	-0.060±0.062	0.009±0.038
Drying	-0.121±0.051	0.002±0.016

A) B)

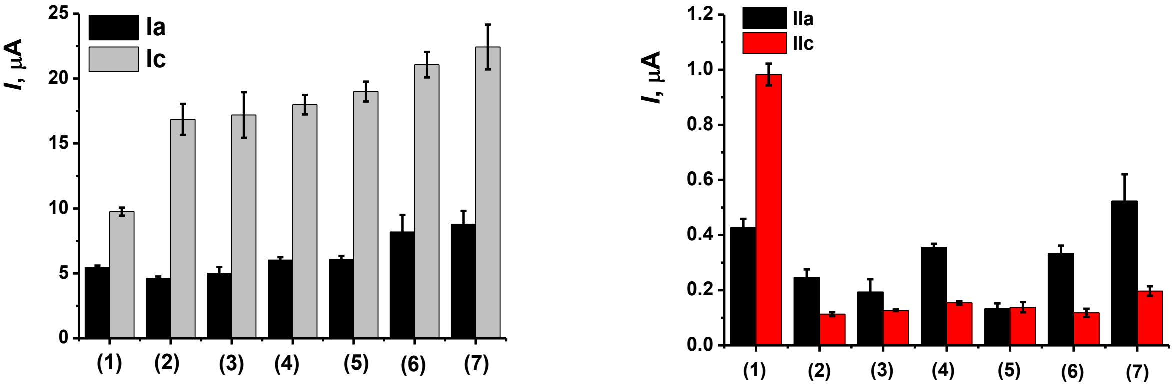

Figure S8. Comparison of peak currents for Ia/Ic (A) and IIa/IIc (B), obtained for SPCE/PNR$_{REL}$ incubated in DNAss solution with different concentrations: (1) no DNA, (2) 100 ng/mL, (3) 1

μg/mL, (4) 10 μg/mL, (5) 100 μg/mL, (6) 1 mg/mL, (7) 2 mg/mL

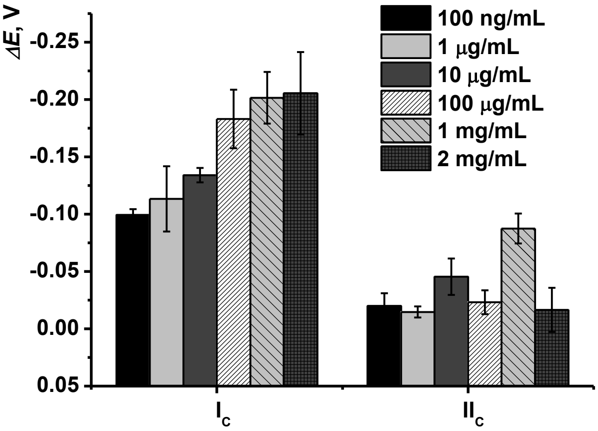

Figure S9. Cathodic potential shift for (Ic) and (IIc) peaks on SPCE/PNR$_{REL}$/DNAss depending on DNAss concentration
